# *AaCycTL* Regulates Cuticle and Trichome Development in Arabidopsis and *Artemisia annua* L.

**DOI:** 10.3389/fpls.2021.808283

**Published:** 2021-12-23

**Authors:** Boran Dong, Xingxing Wang, Rui Jiang, Shiyuan Fang, Jinxing Li, Qing Li, Zong you Lv, Wan sheng Chen

**Affiliations:** ^1^Research and Development Center of Chinese Medicine Resources and Biotechnology, Shanghai University of Traditional Chinese Medicine, Shanghai, China; ^2^Research and Development Center of Chinese Medicine Resources and Biotechnology, The Ministry of Education (MOE) Key Laboratory for Standardization of Chinese Medicines, Institute of Chinese Materia Medica, Shanghai University of Traditional Chinese Medicine, Shanghai, China; ^3^Department of Pharmacy, Changzheng Hospital, Second Military Medical University, Shanghai, China

**Keywords:** cyclins, cuticle development, trichome development, *Artemisia annua*, artemisinin

## Abstract

Artemisinin is an important drug for resistance against malaria. Artemisinin is derived from the glandular trichome of leaves, stems, or buds of the Chinese traditional herb *Artemisia annua*. Increasing the trichome density may enhance the artemisinin content of *A. annua*. It has been proven that cyclins are involved in the development of trichomes in tomato, Arabidopsis, and tobacco, but it is unclear whether the cyclins in *A. annua* influence trichome development. In this study, we showed that *AaCycTL* may regulate trichome development and affect the content of artemisinin. We cloned *AaCycTL* and found that it has the same expression files as the artemisinin biosynthesis pathway gene. We overexpressed *AaCycTL* in Arabidopsis, and the results indicated that *AaCycTL* changed the wax coverage on the surface of Arabidopsis leaves. The trichome density decreased as well. Using yeast two-hybrid and BiFC assays, we show that *AaCycTL* can interact with *AaTAR1*. Moreover, we overexpressed *AaCycTL* in *A. annua* and found that the expression of *AaCycTL* was increased to 82–195%. Changes in wax coverage on the surface of transgenic *A. annua* leaves or stems were found as well. We identified the expression of the artemisinin biosynthesis pathway genes *ADS*, *CYP71AV1*, and *ALDH1* has decreased to 88–98%, 76–97%, and 82–97% in the *AaCycTL*-overexpressing *A. annua* lines, respectively. Furthermore, we found reduced the content of artemisinin. In agreement, overexpression of *AaCycTL* in *A. annua* or Arabidopsis may alter waxy loading, change the initiation of trichomes and downregulate trichome density. Altogether, *AaCycTL* mediates trichome development in *A. annua* and thus may serve to regulate trichome density and be used for artemisinin biosynthesis.

## Highlights

1. *AaCycTL* was shown to regulate trichome development
2. *AaCycTL* interacted with *AaTAR1* modulation of cuticle biosynthesis

## Introduction

Artemisinin, a product of the Chinese medicinal plant *Artemisia annua* L., is an important drug for curing malaria due to its unique 1,2,4-trioxane ring structure. Recently, the usefulness of artemisinin has increased because it has the potential to inhibit *Mycobacterium tuberculosis* (Zheng et al., 2017) and may have a bright future for curing diabetes (Li et al., 2017). This drug also has potential in tumor treatment (Caddeo et al., 2021).

Artemisinin is specifically biosynthesized in the trichomes of leaves, buds, or stems of *A. annua*. Trichomes can usually be divided into glandular trichomes and non-glandular trichomes. The glandular trichome is an important organ that can be used to biosynthesize and store secondary metabolites, such as scents, pigments, and medicinally active compounds (Chalvin et al., 2020). Many factors affect the development of trichomes. The *GLABRA1* (*GL1*)-*GLABRA3* (*GL3*)/ENHANCER OF GLABRA3 (*EGL3*)-TRANSPARENT TESTA GLABRA1 (*TTG1*) complex is the core component of trichome development and may activate the expression of *GL2* and promote trichome development in Arabidopsis (Ishida et al., 2008). In glandular trichome development, HDzip transcription factors are the main regulators of trichome development; moreover, *Wo* is the core regulator of trichome development in tomato, and it can interact with a B-type cyclin gene, *SlCycB2* (Yang et al., 2011). This gene can also interact with *SlMYB31* to promote wax accumulation and directly bind to the promoter of *SlCER6* to affect very-long-chain fatty acid elongation (Xiong et al., 2020). In *A. annua*, *AaHD8* interacts with *AaMIXTA1*, which is involved in cuticle biosynthesis. *AaHD8* can directly bind to the promoter of *AaHD1* to modulate trichome initiation (Yan et al., 2017, 2018; Shi et al., 2018) and thus enhance artemisinin accumulation. *AaMIXTA1* regulates trichome development by inducing the expression of genes in the cuticle biosynthesis pathway (Shi et al., 2018); however, its mechanism is still unclear.

The surface of trichomes is covered by cuticles and wax (Duke and Paul, 1993). The cuticle content may affect the initiation of trichome development (Shi et al., 2018). In addition to modulating the gene expression of the cuticle, which may increase trichome density, cyclins may also have the potential to regulate the number of trichomes developed. Plant growth and development are important biological processes that are involved in the mitotic cell cycle. Cyclins determine the transition among the four distinct phases: the postmitotic interphase (G1), DNA synthesis phase (S), premitotic interphase (G2), and mitosis/cytokinesis phase (M) (Qi and Zhang, 2019). Cyclins can be classified into 10 groups: A-, B-, C-, D-, H-, L-, T-, U-, SDS-, and CycJ18-type cyclins (Vandepoele et al., 2002; Wang et al., 2004). Each group may have different biological functions by regulating the transitions from G1-to-S, S-to-M, and G2-to-M, leading to cell growth and development (Inzé and De Veylder, 2006). Exogenous brassinosteroids were shown to regulate fruit development in cucumber by inducing the expression of cell cycle-related genes, such as *CycA*, *CycB*, *CycD3;1*, and *CycD3;2* (Fu et al., 2008). Cyclin *CYCA3;4* is a postprophase target of APC/C ^CCS52A2^ E3-Ligase and causes disorganized formative cell divisions in Arabidopsis (Willems et al., 2020). *CYCD3* is a key rate-limiting cytokinin response and is involved in cell proliferation and endocycles in Arabidopsis (Dewitte et al., 2007). Cyclins may participate in trichome development. Type B cyclins *CYCLIN B1;2* may trigger cell division and induce mitotic division, leading to the generation of multicellular trichomes in Arabidopsis (Schnittger et al., 2002a). B-type cyclins *SlCycB2*, *SlCycB3*, and *NtCycB2* play negative roles in trichome development in tomato (Gao et al., 2017).

Here, we cloned a cyclin gene, cyclin trichome less (*AaCycTL*), and found that it affects the coverage of cuticles in transgenic Arabidopsis and transgenic *A. annua*. *AaCycTL* can interact with *AaTAR1* to regulate the biosynthesis of cuticle. We also found that *AaCycTL* negatively regulates trichome development and reduces the artemisinin content. These results indicated that *AaCycTL* affects the artemisinin content by modulating the biosynthesis of cuticles.

## Materials and Methods

### Plant Materials and Growth Conditions

Seeds of “Huhao 1” and *Arabidopsis thaliana* were surface-sterilized with 10% sodium hypochlorite (NaClO; v/v) and 0.1% Triton X-100 (v/v) for 5 min and then rinsed three times with sterilized water. These seeds were plated onto a culture plate (90 × 15 mm) with sterile solid Murashige and Skoog (MS) medium. Sterilized seeds planted on the MS medium were transferred to fridge at 4°C for 3 d to synchronize germination. Then, these seeds were transferred to a culture room at 24 ± 2°C under a 16/8 h light/dark photoperiod.

The transgenic plants were transplanted to a soil mixture (vermiculite:perlite:peat moss = 7:0.5:2) with a 16 h light/8 h dark photoperiod with light exposure at 7,500 lux and 26°C.

### Construction and Plant Transformation

The ORF of *AaCycTL* was driven by 35S and generated 35S:*AaCycTL* vectors by the infusion enzyme (Vazyme, China). The 35S:*AaCycTL* constructs were introduced into *Agrobacterium tumefaciens* and used for genetic transformation of Arabidopsis and *A. annua*. The floral dip method was used to transform Arabidopsis (Henriques et al., 2006). For the genetic transformation of *A. annua*, the leaves of 4-week-old sterilized seedlings cultured on MS medium were used as explants. The activated *A. tumefaciens* were centrifuged and suspended in MS liquid medium (containing 100 mm acetosyringone). The leaves were cut off and incubated with *A. tumefaciens* at 20 min, and then leaves were removed from the MS culture and cultured at 28°C in the dark. After 3 d of coculture, the leaves were transferred to shoot culture (MS + 0.5 mg/L 6-BA + 0.05 mg/L NAA + 500 mg/L carbenicillin sodium) and cultured for 3 w with a 16 h light/8 h dark photoperiod and light at 7,500 lux and 26°C. The shoots were transferred to the screening culture (MS + 0.5 mg/L 6-BA + 0.05 mg/L NAA + 50 mg/L hygromycin + 500 mg/L carbenicillin sodium) and cultured for 4 w. Then, these screened seedlings were transferred onto the rooting culture (1/2 MS) for 4 w. The rooted plants were transferred to a soil mixture (vermiculite:perlite:peat moss = 7:0.5:2) for artemisinin detection and gene expression assays.

### Artemisinin Content Assay

*Artemisia annua* leaves were dried at 50°C for 24 h and then ground into powder. Methanol was used as the solvent to extract artemisinin from powder samples (0.1 g). Artemisinin detection was performed with a Waters Alliance 2695 HPLC system (Zhang et al., 2009).

### TB Staining Assay

Arabidopsis and *A. annua* were stained in TB following the method according to the manufacturer’s instructions (Tanaka et al., 2004). The plant materials were planted on MS medium and used for TB staining assays. Different tissues of Arabidopsis and *A. annua* were stained with TB at a concentration of 0.05% (w/v) TB (Sigma, St Louis, MO, United States). After 2 min of staining, the TB solution was washed with pure water to remove excess TB from the plant surface.

### Confocal Microscopic Observation

The full-length ORF of *AaCycTL* was introduced into vectors 35S:YFP, and the constructs 35S:YFP:*AaCycTL* were generated by the infusion enzyme (Vazyme, China). The vectors were introduced into *A. tumefaciens* by the freeze–thaw method (Zhang et al., 2009). The positive clones were cultured at 28°C and 180 rpm until OD600 reached 1.0 with LB culture medium. Then, the supernatant was removed by centrifugation at 4,000 rpm for 5 min, and the pellet was completely suspended in MS liquid medium (containing 10 mm MES and 150 mm acetosyringone) at OD600 = 0.6. After 3 h of preculture, the suspension was injected into 5-week-old tobacco leaves. Confocal laser microscopy (Leica Microsystems, Wetzlar, Germany) was used to observe YFP signaling in tobacco leaves after infiltration for 48–72 h.

### RNA Isolation and qPCR Analysis

Total RNA was extracted from the control and three independent Arabidopsis and *A. annua* transgenic plant lines containing 35S:*AaCycTL*. The extraction method was performed according to the manufacturer’s instructions for the Tiangen kit (China, Beijing). The concentration of total RNA was tested by a NanoDrop 2000 Spectrophotometer (Thermo Scientific) and then converted to cDNA by a PrimeScript^™^ RT Master MIX kit (Takara-Bio, Dalian, China).

According to the manufacturer’s instructions, reverse transcription was performed using a PrimeScript RT kit (Takara) with a total reaction volume of 20 μl and 1 μg of total RNA. The steps of the reverse transcription were as follows: 70°C for 3 min, 42°C for 30 min, and 80°C for 15 s. The synthesized cDNA was diluted and mixed with SYBR Green (Takara-Bio, Dalian, China) according to the manufacturer’s instructions, followed by a thermal cycling profile: 95°C for 15 min, 40 cycles of 95°C for 15 s, 58°C for 30 s, and 72°C for 20 s. The ΔΔCt method was used to calculate the gene expression, and the actin gene was used as the endogenous control.

### Scanning Electron Microscopy

Tissues were collected and fixed with 2.5% (v/v) glutaraldehyde in phosphate buffer (pH 7.4) at 4°C for 12 h, and then samples were rinsed 4 × 15 min. All the samples were dehydrated at alcohol concentrations of 50, 60, 70, 80, 95, and 100% for 3 min. The dehydrated samples were dried by a critical point drying device (Leica EM CPD030). Following coating with gold particles, tissues were observed by scanning electron microscopy (SEM; Nova NanoSEM 230; FEI Company, Hillsboro, OR, United States; Shi et al., 2018).

### Phylogenetic Tree and Global Expression of the Cyclin Family

The *A. annua* cyclin family was identified from our previous genome information (Shen et al., 2018) using BLASTP queries with known Arabidopsis cyclins. The transcriptome data of different parts of *A. annua* (SRR019547 for mature leaf trichomes; SRR019254 for meristems; SRR019548 for bud trichomes; SRR019549 for cotyledons; SRR019546 for young leaf trichomes) were downloaded from the Sequence Read Archive database of the NCBI^1^ (Graham et al., 2010). Cyclin reads of different parts (cotyledons, meristems, young leaves, mature leaves, and buds) were used for heatmap analysis (Shi et al., 2018).

MultiExperiment Viewer v.4.9.0 was used for hierarchical cluster analysis of the heatmap (Zhang et al., 2015). The phylogenetic tree was built by MEGA7 software,^2^ and the cyclin sequence alignment was analyzed by CLUSTALX (Trinity College, Dublin, Ireland).

### Trichome Density Counting

The glandular trichomes exhibited autofluorescence and were counted with a fluorescence microscope. The mature leaves of *A. annua* (leaf 6, the sixth leaf below the apical meristem) were collected and used for trichome counting (Yan et al., 2017). The glandular trichomes on the axial side of the leaf were photographed with a microscope (Olympus, BX43) and counted with IMAGEJ software. The leaves were from three different independent transformants, and the same part was used to count the density of trichomes.

## Results

### Cyclin Characteristics

**Figure 1:**
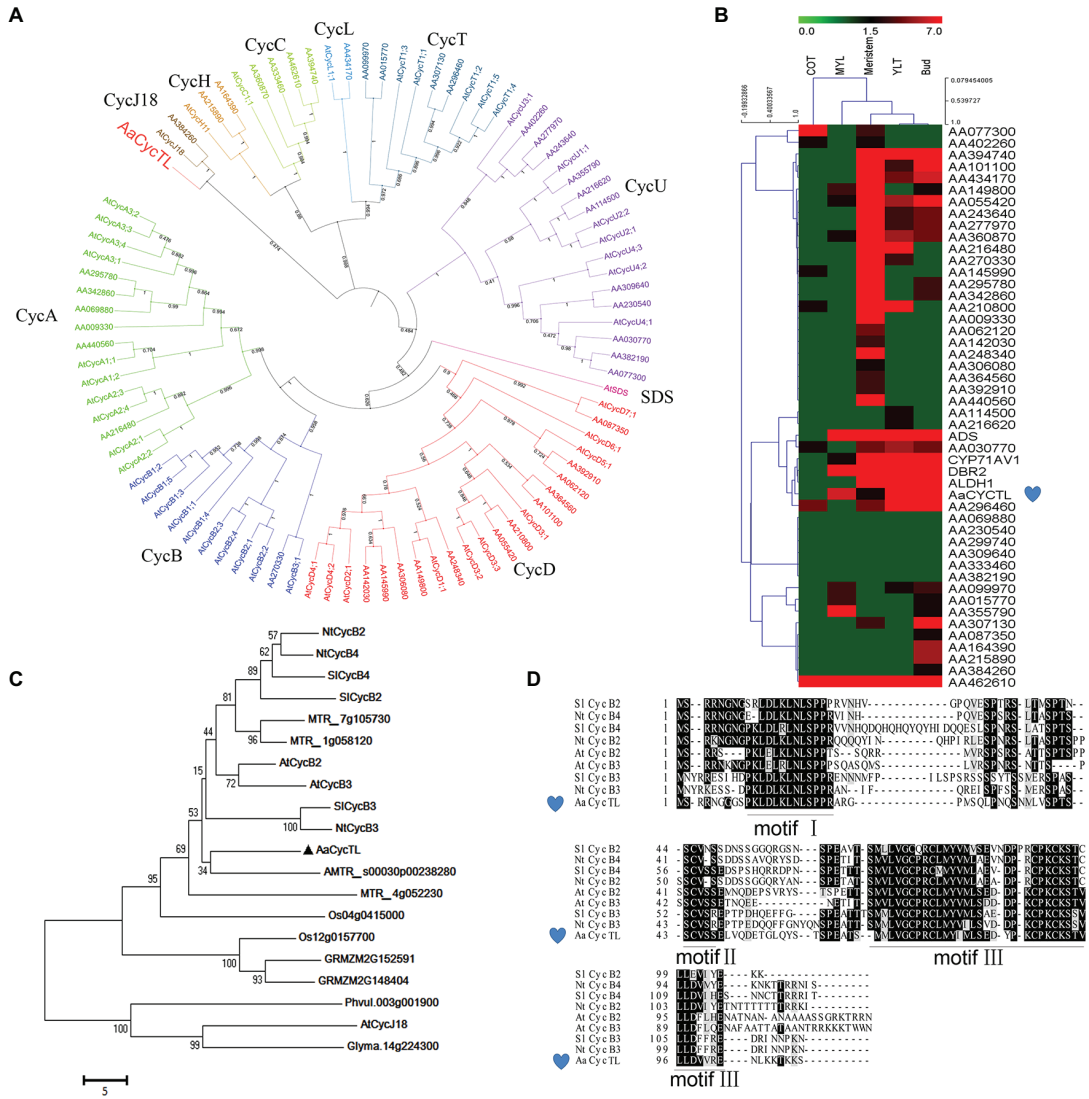
Characteristics of *AaCycTL*. **(A)** Phylogenetic tree analysis of cyclins between Arabidopsis and *Artemisia annua*. The phylogenetic tree was constructed by MEGA 7 with 1,000 bootstrap replicates of the neighbor-joining method. iTOL was employed to embellish the phylogenetic tree. Cyclins can be classified into 10 groups, A-, B-, C-, D-, H-, L-, T-, U-, SDS-, and CycJ18-type cyclins, and different cyclins are marked with different colors. **(B)** The heatmap represents the gene expression files of cyclins in different tissues (cotyledon, COT; mature leaves, MYL; young leaves, YLT) of *A. annua*. The red, black, and blue solid bars represent high, middle, and low levels of gene expression, respectively. *ADS*, *CYP71AV1*, *DBR2* and *ALDH1* are marker genes. **(C)** The phylogenetic tree indicated that *AaCycTL* is in the same clade as AMTR_s00030p00238280. Bootstrap values are 1,000 trials. **(D)** Protein alignment of *AaCycTL* and other closest homologs in Arabidopsis, tobacco, and tomato. The motifs I, II, III of the cyclins are highly conserved.

To investigate cyclin genes in *A. annua*, we annotated the gene information and chose the cyclin genes to build a phylogenetic tree according to our genome information (Shen et al., 2018). In Arabidopsis, 10 classes (A, B, C, D, H, L, T, U, SDS, and CycJ18-type) of cyclins have been described (Wang et al., 2004). The sequence of cyclins is not conserved, and with relatively low sequence similarity, we used the Arabidopsis cyclin sequence as the reference. We found 9 classes of cyclins in *A. annua* and an absence of SDS cyclin (Figure 1A). Therefore, no cyclins were obviously up- or downregulated in *A. annua*.

We analyzed the expression patterns of the cyclins in *A. annua via* a heatmap. Figure 1B shows that *AaCycTL* clustered with the same clade of artemisinin biosynthetic pathway genes, such as *ADS*, *CYP71AV1*, *DBR2*, and *ALDH1*, four of them are mainly expressed in glandular trichomes in *A. annua* and participate in artemisinin biosynthesis. Regulators of trichomes may have the same expression files as artemisinin biosynthesis genes. Therefore, these results indicated that *AaCycTL* was involved in artemisinin biosynthesis or trichome development. The results of amino acid sequence BLAST indicated that AMTR_s00030p00238280 is the homolog of *AaCycTL* (Figure 1C). Multiple sequence alignment analysis shows that *AaCycTL* is the same as other cyclins with three conserved motifs (Figure 1D). These results suggest that *AaCycTL* has unique biological functions in *A. annua*.

### Expression Profiling of *AaCycTL* and Its Protein Subcellular Localization

**Figure 2:**
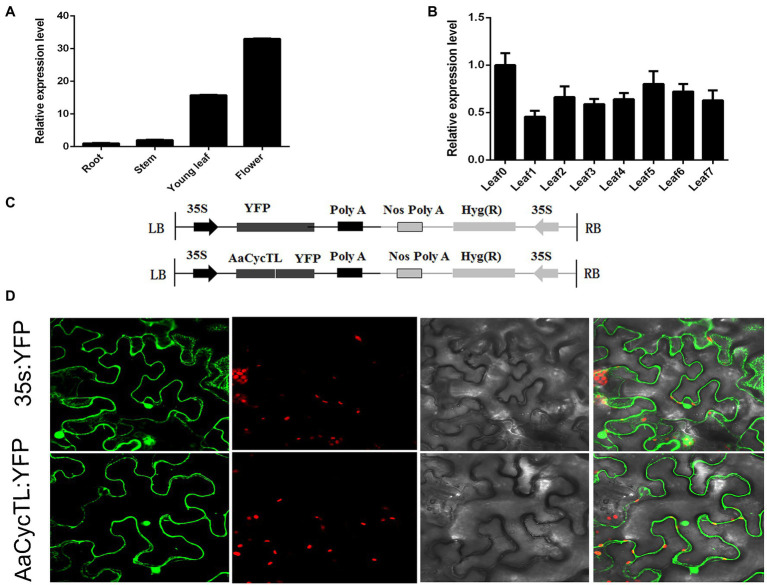
The expression patterns of *AaCycTL* and its protein subcellular localization. **(A)** *AaCycTL* has different expression levels in the roots, stems, leaves, and buds. **(B)** *AaCycTL* has different expression levels in different leaves. **(C)** Diagrams of 35S:*AaCycTL*-YFP constructs. **(D)** The *AaCycTL* protein is localized in the cytoplasm and nucleus of tobacco leaves by transient expression. The 35S:YFP gene was used as control.

To evaluate the underlying functions of *AaCycTL* in *A. annua* growth and development, we detected the expression patterns of *AaCycTL* in roots, stems, young leaves, and mature leaves. *AaCycTL* was mainly expressed in young leaves and buds (Figures 2A,B) and had the same expression profiles as trichome development genes, indicating that *AaCycTL* may be involved in the development of trichomes.

The subcellular localization of the protein may affect the function of the gene (Yu et al., 2006). To investigate the subcellular localization of *AaCycTL*, we fused *AaCycTL* with YFP and injected it into tobacco leaves mediated by *A. tumefaciens*. The control 35S::YFP was located in the cytoplasm and cell nucleus, and *AaCycTL* was located the same as the control (Figure 2C), indicating that *AaCycTL* has the same subcellular localization as transcription factors. We deduced that *AaCycTL* may interact with transcription factors to perform its functions (Figure 2D).

### *AaCycTL* Changes Cuticular Wax Coverage and Composition on Arabidopsis Leaves

**Figure 3:**
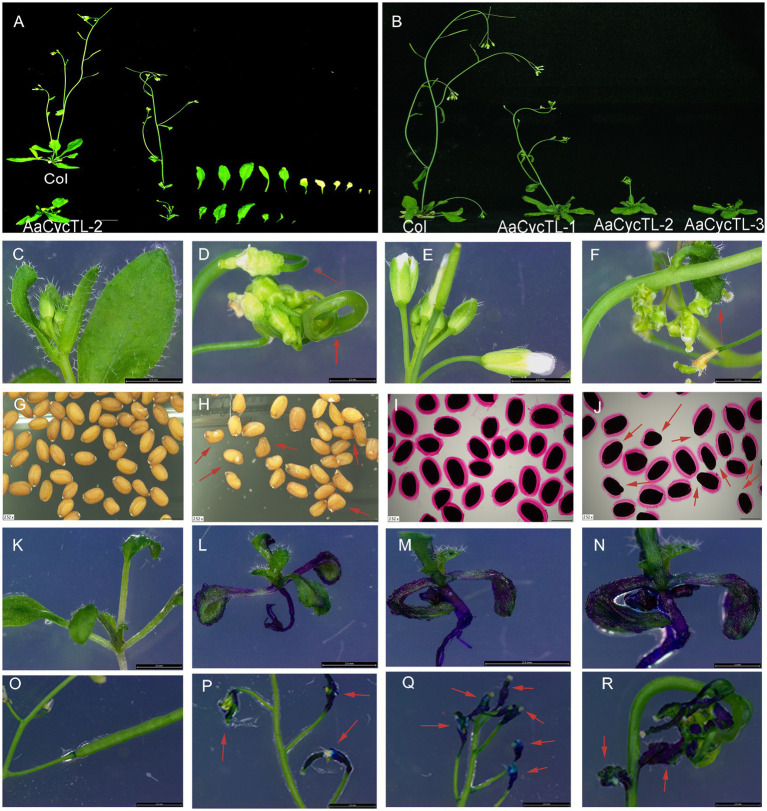
Phenotypes of *AaCycTL*-overexpressing Arabidopsis. Transgenic plants show fewer leaves **(A)** and less biomass **(B)**, curly leaves **(D)**, curly fruit pods **(F)**, abnormal seeds **(H)**, defection of mucilage secretory cells by ruthenium red staining **(J)**, changes in the cuticular wax coverage on the leaves by TB staining assay **(L–N)**, and changes in cuticular wax coverage on the fruit pod **(P–R)** by TB staining assay when contrasted with the Col. **(C)** normal leaves of Col, **(E)** normal flowers and fruit pods of Col, **(G)** normal seeds of Col, **(I)** seeds of Col stained by ruthenium red, **(K,O)** different development stage of Col stained by TB. Red arrows represent the obvious phenotype tissue.

To test the gene function of *AaCycTL* in Arabidopsis, we overexpressed *AaCycTL* in Arabidopsis *via* the floral dip method (Zhang et al., 2006). We detected the expression of *AaCycTL* in transgenic plants and found that *AaCycTL* expression increased manyfold (Supplementary Figure S1), and transgenic plants showed obvious phenotypes with relatively low biomass (Supplementary Figure S2), curled leaves, twisted stems, shortened fruit pods, and few leaves and seeds (Figures 3A–F). We also detected the seed coat mucilage of transgenic seeds because the appearance of seeds changed. Ruthenium red (RR) staining of the seeds indicated that the transgenic seeds showed defective mucilage compared to Col (Figures 3G–J).

**Figure 4:**
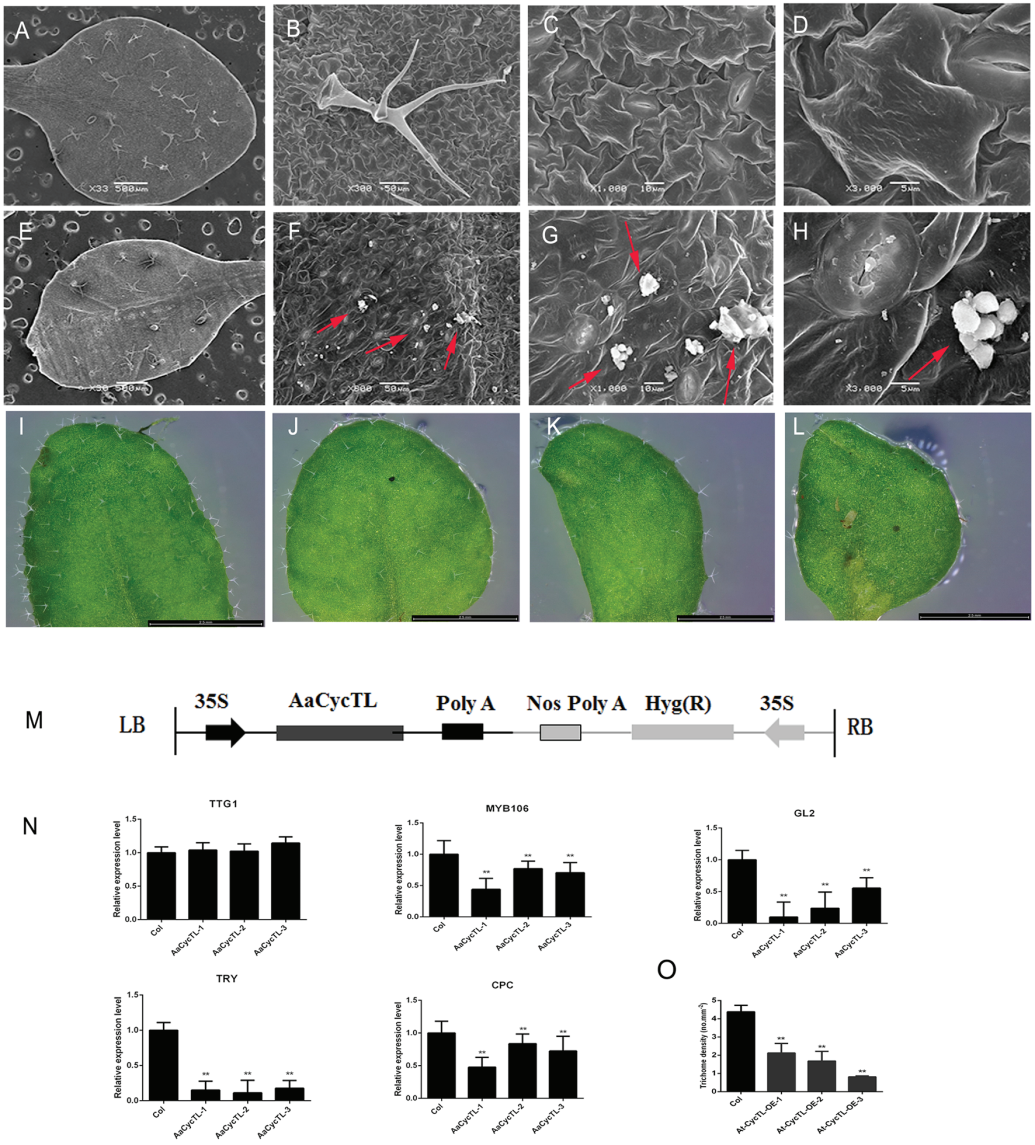
Phenotypes and gene expression of *AaCycTL*-overexpressing Arabidopsis. **(A–D)** Control phenotype of col under SEM observation. **(E–H)** Waxy crystals on the surface of genetically modified plants. Red arrow means waxy crystals particles. **(I–L)** The trichomes of transgenic Arabidopsis leaves changed. **(M)** Diagrams of 35S:*AaCycTL* constructs. **(N)** The expression of *AaCycTL*, *MYB106*, *TTG1*, *GL2*, *TRY*, and *CPC* in the transgenic plants. **(O)** The trichome density of *AaCycTL*-overexpressing Arabidopsis. ^**^p < 0.01, Student’s *t*-test.

*Cycb3* in tomato plays an important role in cuticular biosynthesis (Gao et al., 2017), so we deduced that *AaCycTL* may participate in cuticular biosynthesis. To further substantiate this finding, we investigated whether *AaCycTL*-overexpressing plants can be stained by TB. The young leaves of the seedlings and the fruit pods were easy to stain (Figures 3K–R), and we deduced that the array of cuticles on the leaves of the seedlings or on the young fruit pods was disordered. Therefore, we employed SEM to observe the cuticles on the leaves of Arabidopsis. The results show that some waxy crystal particles were formed on the surface of the leaves in the transgenic plants (Figures 4A–H). To further study whether *AaCycTL* regulates cuticular biosynthesis, we tested the expression of genes involved in cuticular biosynthesis by qPCR, the results of which showed that the expression of *AaCycTL* increased in the transgenic plants and that the expression of *MYB106* was decreased several-fold (Figure 4N).

The biosynthesis of waxy compounds may affect the initiation of trichomes (Li et al., 2021), so we investigated the expression of genes involved in trichome development. We detected the *GL2*, *TTG1*, *TRY*, and *CPC*. The expression level of *GL2* was decreased, while that of *TTG1* was unchanged. We also detected the expression of negative trichome regulators, such as *TRY* and *CPC*. The results indicated that the expression of *TRY* and *CPC* was reduced several-fold (Figure 4M). Finally, we counted the trichome density in Arabidopsis. The trichome density of transgenic plants was decreased compared with that of Col plants (Figures 4I,O). Therefore, the results show that *AaCycTL* downregulated the trichome number by affecting waxy coverage in Arabidopsis.

### *AaCycTL* Interacts With *AaTAR1*

**Figure 5:**
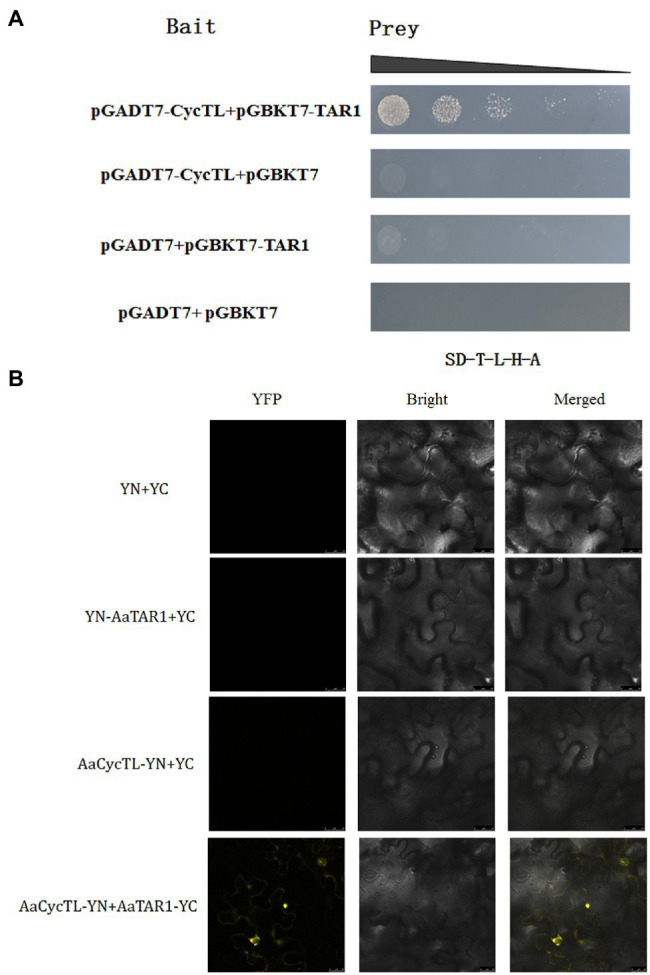
*AaCycTL* interacted with *AaTAR1* in yeast cells and tobacco leaves. **(A)** Y2H indicated that *AaCycTL* interacts with *AaTAR1*. The bait and prey plasmids were co-transformed into yeast and detected on SD medium lacking Trp, Leu, His, and Ade. **(B)** A BiFC assay was used to test the interactions between *AaCycTL* and *AaTAR1*. *AaCycTL* was fused to the N-terminus of YFP, and *AaTAR1* was fused to the C-terminus of YFP.

Previous studies indicated that *TAR1*, a homologous gene of *AtWIN1* and *AtSHINE3* in *A. annua*, participates in wax load and cuticle permeability (Tan et al., 2015). To elucidate the possible mechanism by which *AaCycTL* regulates cuticular wax coverage, we detected the interaction between *AaCycTL* and *AaTAR1*. The yeast two-hybrid (Y2H) assay results indicated that *AaCycTL* interacted with *AaTAR1* (Figure 5A). To further test the interaction between *AaCycTL* and *AaTAR1*, we employed the biomolecular fluorescence complementation (BiFC) method. In Figure 5B, *AaCycTL* can interact with *AaTAR1*. Another gene, *AaMIXTA1*, was shown to be involved in cuticular biosynthesis in *A. annua* (Tan et al., 2015). However, the Y2H assay results indicated that there was no interaction between *AaCycTL* and *AaMIXTA1*. Thus, *AaCycTL* may regulate the cuticle by interacting with *AaTAR1*.

### *AaCycTL* Affects Trichome Distribution in *A. annua*

**Figure 6:**
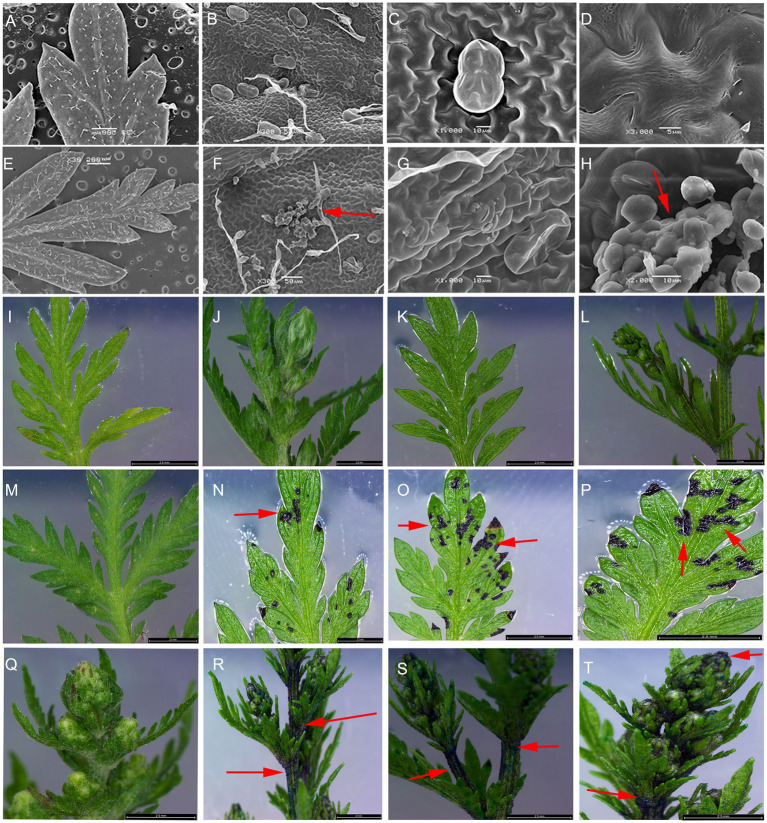
Cuticular wax coverage changed in *AaCycTL*-overexpressing *A. annua*. **(A–H)** The waxy array was changed on the surface of the transgenic plant leaves. Crystal waxy particles were found on the surface of the transgenic plant leaves. The red arrow means the crystal waxy particles. **(I,M,Q)** control tissues. **(J–L)** The nontransgenic plants were stained with TB. **(N–P,R–T)** Leaves and buds were stained by TB.

To investigate the gene function of *AaCycTL* in *A. annua*, we overexpressed *AaCycTL* in *A. annua*, which was driven by the 35S promoter. *AaCycTL* can regulate cutin biosynthesis in Arabidopsis, so we deduced that *AaCycTL* may participate in cutin biosynthesis in *A. annua*. The SEM results show that the surface of the transgenic plant leaves formed some waxy crystals (Figures 6A–H). To further investigate abnormal cuticular wax loading, we used the TB test to assess cuticular wax coverage. The results indicated that *AaCycTL* changed cuticular wax coverage on the leaves and stems of the transgenic plants compared with those of the control (Figures 6I–T).

**Figure 7:**
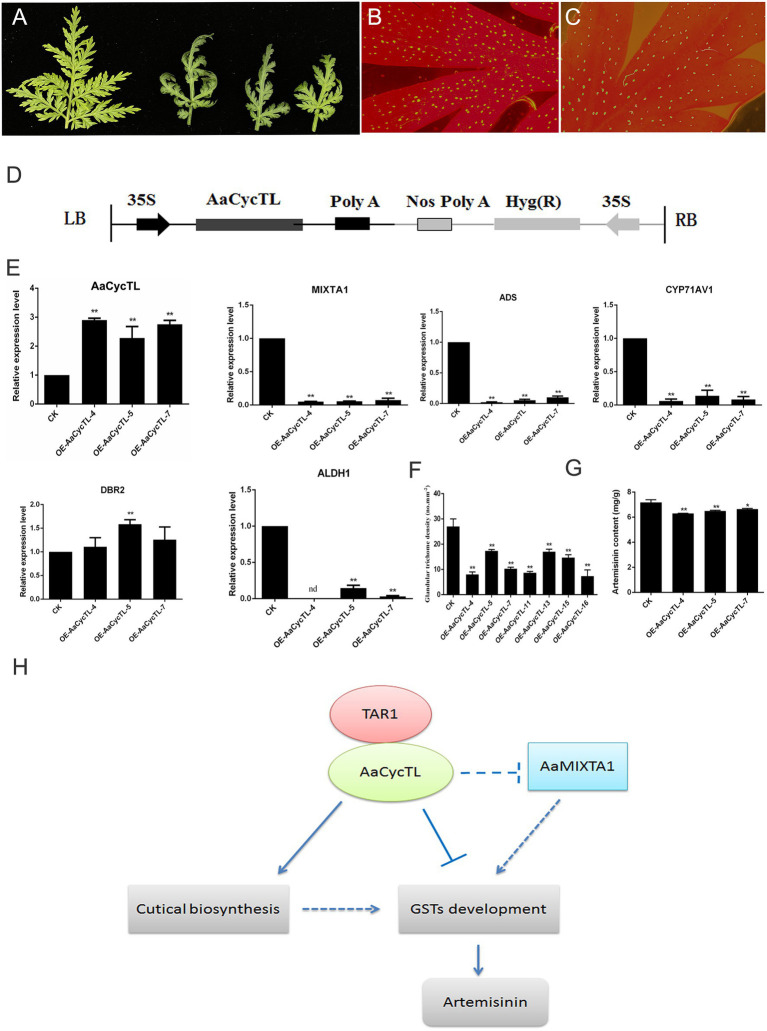
**(A)** Phenotypes of the *AaCycTL*-overexpressing *A. annua* leaves. The trichome density on the leaves of the control **(B)** and the transgenic plants **(C)**. **(D)** Diagrams of 35S:*AaCycTL* constructs. **(E)** The expression of *AaCycTL*, *MIXTA1*, *ADS*, *CYP71AV1*, *DBR2*, and *ALDH1*. **(F)** The trichome number of the *AaCycTL*-overexpressing plants. **(G)** The artemisinin content in the transgenic plants. ^*^p < 0.05, ^**^p < 0.01, Student’s *t*-test. **(H)** The model of *AaCycTL* interaction with *AaTAR1* to regulate trichome development. *AaCycTL* negatively regulates the expression of *MIXTA1*, which is an important transcription factor that modulates trichome development. *AaCycTL* and *AaTAR1* may coregulate the biosynthesis of cuticular trichomes, which may affect the initiation of glandular trichomes.

The phenotype of the transgenic plants changed, and the transgenic plants showed curled leaves and fewer trichomes than the control plants (Figures 7A–C). We detected the gene expression of *AaCycTL*, and the results indicated that the mRNA of *AaCycTL* was increased several-fold (Figure 7D). qPCR was employed to detect the expression of trichome-related genes in *A. annua*. The expression of *AaMIXTA1* is decreased in Figure 7E, indicating that *AaCycTL* has a regulatory effect on cutin and trichome development. Therefore, we detected the trichome number in transgenic *A. annua*. The results showed that the trichome density in transgenic plants decreased when compared with that in control plants (Figure 7F).

### *AaCycTL* Affects the Artemisinin Content

A previous study indicated that trichome density is positively related to artemisinin content (Yan et al., 2018). The results of this study show that *AaCycTL* regulates trichome density, and we hypothesized that *AaCycTL* may affect the biosynthesis of artemisinin. To this end, the expression of artemisinin biosynthesis pathway genes was detected by qPCR, and β-actin was used as the interference gene. The qPCR results indicated that the mRNA levels of the artemisinin biosynthesis genes *ADS*, *CYP71AV1*, and *ALDH1* were all downregulated in the transgenic plants, while the expression of *DBR2* was upregulated in the transgenic plants (Figure 7E). We deduced that the artemisinin content may be downregulated in the transgenic plants. Therefore, we investigated the content of artemisinin in transgenic plants and the control. The content of artemisinin was lower in transgenic plants than in control plants (Figure 7G). All the results suggest that *AaCycTL* interacted with *AaTAR1*, both of which can affect cuticular biosynthesis and trichome initiation, leading to an effect on the biosynthesis of artemisinin (Figure 7H).

## Discussion

Glandular trichome is an important tissue that stores and synthesizes secondary metabolites, such as scents, pigments, and medicinally active compounds (Chalvin et al., 2020). Modulation of the density of trichomes is an important method to increase the content of secondary metabolites (Yan et al., 2017, 2018; Shi et al., 2018). Cyclins are important regulators that modulate trichome development (Yang et al., 2011). Trichome development is a complex issue involving many factors, such as phytohormones (Maes et al., 2011; Li et al., 2021), transcription factors (Shi et al., 2018; Yan et al., 2018; Xie et al., 2021), and cyclins (Schnittger et al., 2002a; Gao et al., 2017).

### Cuticular Wax Coverage and Composition Affects Trichome Development

The waxy cuticle can cover all epidermal tissues, including trichomes and leaves. The composition of wax on the trichome surface is different from that of other epidermal cells (Hegebarth et al., 2016). Wax from isolated trichome is composed of four compound classes. Alkanes are the main component of the mixture, (unbranched) primary n-alcohols, branched primary alcohols and alkenes are the minor components (Hegebarth et al., 2016). Trichome development is involved in cutin/wax deposition (Chalvin et al., 2020). The surface of trichomes is covered with waxy cutin. Sticky peel mutants of tomato lead to cutin deficiency, alter the wax profile, and reduce glandular trichome density (Nadakuduti et al., 2012). Inhibition of the AP2/ERF transcription factor *AaTAR1* may alter cutin/wax deposition. In *TAR1*-RNAi transgenic plants, the leaves are covered with abnormal waxy deposits, which suggests that the permeability of the cuticle is enhanced and leads to defects in the synthesis of aliphatic components. Abnormal waxy deposits lead to abnormal development of trichomes (Tan et al., 2015). Overexpression of *AaMIXTA1* in *A. annua* increased the total wax compound content by 10–40% in transgenic plants, including that of cuticular wax, β-amyrin, hexacosanoic acid, docosanoic acid, and hexacosanol. At the same time, the total cutin monomer load on the surface of mature leaves was increased by 20–25% in transgenic plants, including that of 10,16-diOH-hexadecanoic, 16-OH-hexadecanoic, and monofunctional hexadecanoic acids. Therefore, changes in the wax content of trichomes may affect their development (Shi et al., 2018). *MYB16* and *MYB106* are the main regulators of cutin coverage in Arabidopsis, and both of these genes affect trichome development (Oshima and Mitsuda, 2014). We found that *AaCycTL* may affect cuticular wax coverage on trichomes of Arabidopsis and *A. annua* leaves. In the transgenic Arabidopsis and *A. annua* plants, many waxy crystals were observed on the leaves (Figures 4F–H, 6F–H), indicating that *AaCycTL* may change the loading of waxy compounds. Therefore, the trichome density was decreased in the transgenic Arabidopsis and *A. annua* plants compared to the control plants (Figures 4J–L, 7C). *AaCycTL* plays the same roles as transcription factors, such as *AaTAR1*, *AaMIXTA1*, *MYB16*, and *MYB106*; however, we did not find transcription factors that directly bind to the promoter of *AaCycTL*. *AaCycTL* can interact with *AaTAR1* to regulate the coverage of waxy compounds (Figure 5). To date, there is no research report on the interaction of cyclin with the AP2/ERF transcription factor to regulate wax biosynthesis, and the modulation of the expression of *AaCycTL* remains unclear.

### Regulation of Trichome Development by Cyclins

Cyclins are important regulators of plant growth and development (Inzé and De Veylder, 2006). In mitotic cell cycle progression, the S phase is involved in trichome development (Ishida et al., 2008). Therefore, modulation of the G1-to-S transition may trigger DNA replication and mitosis, leading to the development of trichomes (Ishida et al., 2008).

Previously, some cell cycle-related proteins were shown to exhibit trichome development functions. *CYCLIN B1;2* can promote the transition of G2 to M phase and induce a shift from endoreduplication to mitosis. Under the influence of the GL2 promoter, *CYCLIN B1;2* can generate multicellular trichomes (Schnittger et al., 2002a). *CYCD3;1* plays the same role as *CYCLIN B1;2*, promoting the G2-to-M phase transition. The promoter of GL2 drives *CYCD3;1* to obtain multicellular trichomes (Schnittger et al., 2002b). SIAMESE (SIM), a cyclin-dependent kinase (CDK) inhibitor, represses CDK complexes and promotes endoreplication in Arabidopsis trichomes (Wang et al., 2020). CCS52A1 and SIM may cooperate to inhibit the progression of mitotic cyclins (Kasili et al., 2010). Cyclins may interact with trichome regulators to affect trichome density. The B-type cyclin gene *SlCycB2* can interact with *Wo* by physical interaction, and *Wo* is a positive regulator of trichome development (Yang et al., 2011). However, the mechanism between *SlCycB2* and the *Wo* interaction is still unknown. *SlCycB2* may directly affect the wax coverage and composition of trichomes (Gao et al., 2017), or *SlCycB2* may affect the transcriptional activity of wo (Yang et al., 2011). We found that *AaCycTL* interacted with the cuticle regulator *AaTAR1* (Figure 5), which plays important roles in cuticle biosynthesis in *A. annua*. Cyclins may affect the expression of genes involved in trichome development. *TRY* is a negative regulator of trichome development. More trichomes were observed in the *TRY* mutant than in control plants, possibly because *TRY* particles are endoreplicated (Schellmann et al., 2002).

AaCycTL alters the coverage of waxy on the surface of leaves or stems in transgenic plants and plays a negative role in trichome development. The RNAi or Cas9 technology will be used to attenuate the expression of *AaCycTL*, leading to enhance trichome density. Therefore, inhibition of *AaCycTL* in *A. annua* may have a promising future for obtaining high yield artemisinin plant.

## Data Availability Statement

The datasets presented in this study can be found in online repositories. The names of the repository/repositories and accession number(s) can be found in the article/Supplementary Material.

## Author Contributions

WC and ZL conceived and designed the entire research plans and helped with the organization and editing. ZL, BD, XW, and RJ performed most of the experiments. SF provided technical assistance. ZL, JL, WC, and QL wrote the manuscript. All authors contributed to the article and approved the submitted version.

## Funding

This work was funded by the National Natural Science Foundation of China (32070332) and the Shanghai Natural Science Foundation in China (20ZR1453800), Shanghai local Science and Technology Development Fund Program guided by the Central Government (YDZX20203100002948), and National Key R&D Program of China (2019YFC1711100).

## Conflict of Interest

The authors declare that the research was conducted in the absence of any commercial or financial relationships that could be construed as a potential conflict of interest.

## Publisher’s Note

All claims expressed in this article are solely those of the authors and do not necessarily represent those of their affiliated organizations, or those of the publisher, the editors and the reviewers. Any product that may be evaluated in this article, or claim that may be made by its manufacturer, is not guaranteed or endorsed by the publisher.

## Footnotes

^1^ https://trace.ncbi.nlm.nih.gov/Traces/sra/sra.cgi

^2^ https://www.megasoftware.net

## Supplementary Material

Supplementary Figure S1 Expression of AaCycTL in Arabidopsis.

Supplementary Figure S2 The biomass of the AaCycTL-overexpressed plants. ^**^*p* < 0.01, Student’s *t*-test.
