# Supplementary figures and images for: AaCycTL Regulates Cuticle and Trichome Development in Arabidopsis and Artemisia annua L
Source: Front Plant Sci. 2021 Dec 23;12:808283. doi: 10.3389/fpls.2021.808283 (PMC8733389; doi:10.3389/fpls.2021.808283)

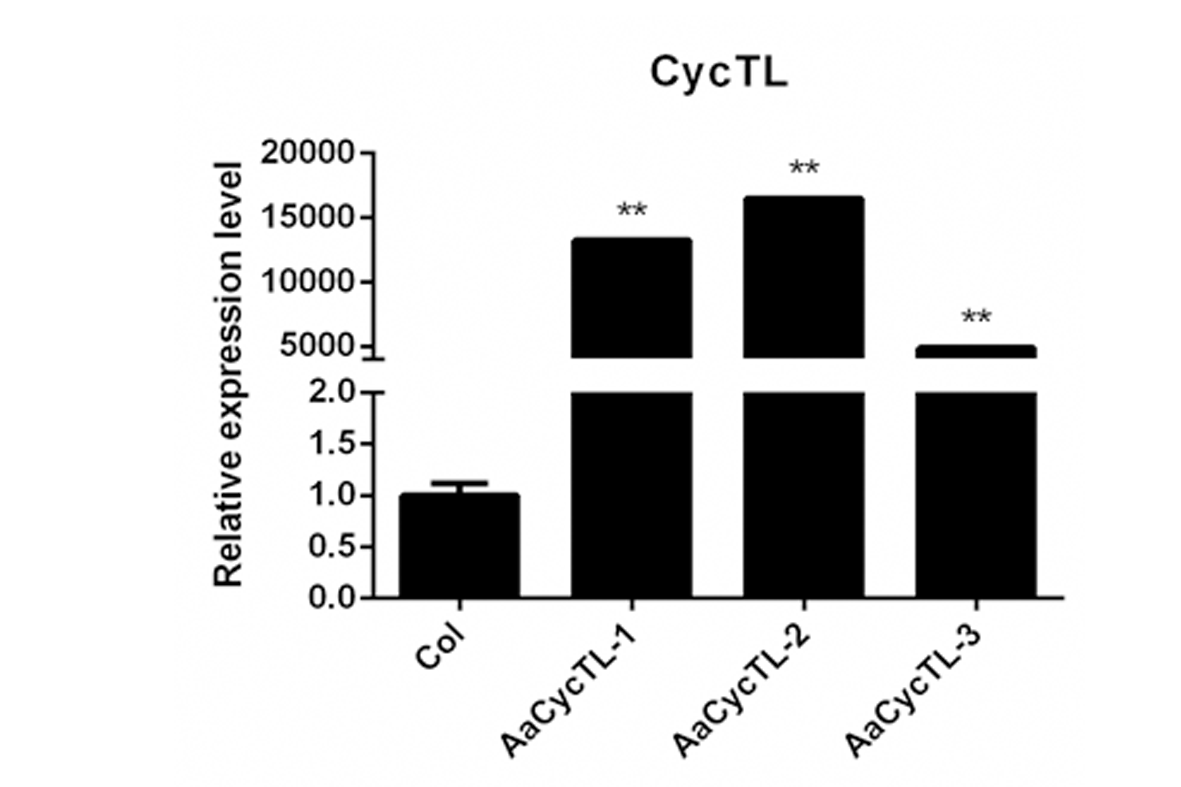

Supplement: Supplementary Figure S1 — Expression of AaCycTL in Arabidopsis. [file Image_1.TIF]

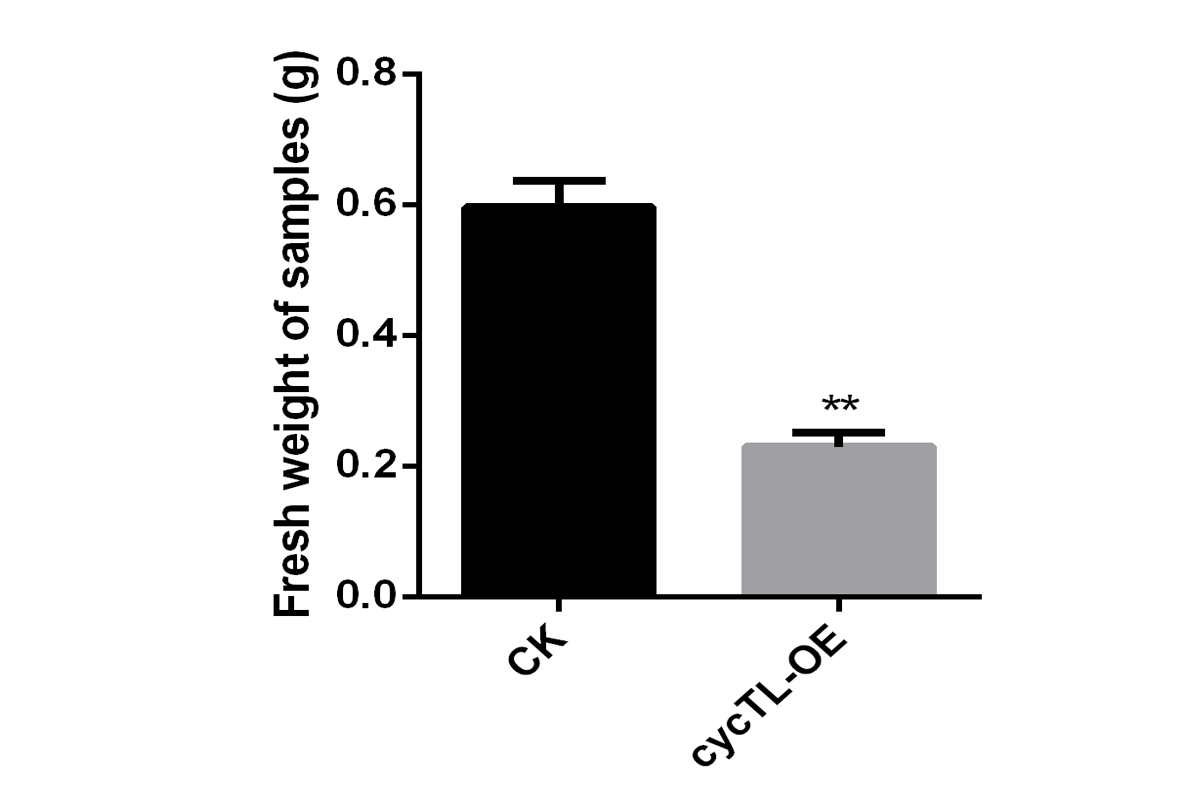

Supplement: Supplementary Figure S2 — The biomass of the AaCycTL-overexpressed plants. **p < 0.01, Student’s t-test. [file Image_2.TIF]
